# Efficient and Accurate Extracting of Unstructured EHRs on Cancer Therapy Responses for the Development of RECIST Natural Language Processing Tools: Part I, the Corpus

**DOI:** 10.1200/CCI.19.00147

**Published:** 2020-05-04

**Authors:** Yalun Li, Yung-Hung Luo, Jason A. Wampfler, Samuel M. Rubinstein, Firat Tiryaki, Kumar Ashok, Jeremy L. Warner, Hua Xu, Ping Yang

**Affiliations:** ^1^ Department of Health Sciences Research, Mayo Clinic, Scottsdale, AZ; ^2^ Division of Pulmonary & Critical Care Medicine, West China Hospital, Sichuan University, Chengdu, Sichuan, China; ^3^ Department of Chest Medicine, Taipei Veterans General Hospital, Taipei City, Taiwan; ^4^ Division of Biomedical Statistics and Informatics, Department of Health Science Research, Mayo Clinic, Rochester, MN; ^5^ Department of Medicine, Division of Hematology/Oncology, Vanderbilt University, Nashville, TN; ^6^ School of Biomedical Informatics, The University of Texas Health Science Center at Houston, Houston, TX; ^7^ Department of Health Sciences Research, Mayo Clinic, Scottsdale, AZ; ^8^ Department of Biomedical Informatics, Vanderbilt University, Nashville, TN

## Abstract

**PURPOSE:**

Electronic health records (EHRs) are created primarily for nonresearch purposes; thus, the amounts of data are enormous, and the data are crude, heterogeneous, incomplete, and largely unstructured, presenting challenges to effective analyses for timely, reliable results. Particularly, research dealing with clinical notes relevant to patient care and outcome is seldom conducted, due to the complexity of data extraction and accurate annotation in the past. RECIST is a set of widely accepted research criteria to evaluate tumor response in patients undergoing antineoplastic therapy. The aim for this study was to identify textual sources for RECIST information in EHRs and to develop a corpus of pharmacotherapy and response entities for development of natural language processing tools.

**METHODS:**

We focused on pharmacotherapies and patient responses, using 55,120 medical notes (n = 72 types) in Mayo Clinic’s EHRs from 622 randomly selected patients who signed authorization for research. Using the Multidocument Annotation Environment tool, we applied and evaluated predefined keywords, and time interval and note-type filters for identifying RECIST information and established a gold standard data set for patient outcome research.

**RESULTS:**

Key words reduced clinical notes to 37,406, and using four note types within 12 months postdiagnosis further reduced the number of notes to 5,005 that were manually annotated, which covered 97.9% of all cases (n = 609 of 622). The resulting data set of 609 cases (n = 503 for training and n = 106 for validation purpose), contains 736 fully annotated, deidentified clinical notes, with pharmacotherapies and four response end points: complete response, partial response, stable disease, and progressive disease. This resource is readily expandable to specific drugs, regimens, and most solid tumors.

**CONCLUSION:**

We have established a gold standard data set to accommodate development of biomedical informatics tools in accelerating research into antineoplastic therapeutic response.

## INTRODUCTION

An electronic health record (EHR) is a digital form of a patient’s medical history, making real-time, patient-centered information available instantly and securely to authorized users. Although primarily designed for billing and to document medical and treatment histories of patients, EHR data can be used for other purposes. Recently, EHRs have been successfully implemented in the majority of US health care systems in various platforms, enabling a surge in secondary uses of EHRs, especially for research. However, because EHRs are created primarily for nonresearch purposes, derived data sets are enormous, crude, heterogeneous, incomplete, and largely unstructured, presenting challenges to effective analyses for timely new and reliable findings. Particularly, research dealing with unstructured clinical notes relevant to patient care and outcome, such as response to therapy, had been ineffectively conducted before the era of artificial intelligence (AI) techniques, due to the complexity of data extraction and accurate annotation. These challenges are being surmounted with the application of AI techniques (eg, clinical natural language processing [NLP] tools and machine learning)^1,2^; as a consequence, EHRs are gradually being used to facilitate and accelerate research relevant to patient care.^3^

**CONTEXT**

**Key Objective**
To identify textual sources for the widely used RECIST information in electronic health records (EHRs) and develop a fully annotated gold standard corpus of anti-lung cancer pharmacotherapy and response entities.
**Knowledge Generated**
A data corpus was developed with pharmacotherapies and the four RECIST response levels of complete response, partial response, stable disease and progressive disease, starting with a total of 55,120 medical notes. A gold standard corpus was built containing 609 randomly selected lung cancer cases (n = 503 for training and n = 106 for validation) and 736 fully annotated clinical notes (n = 617 for training and n = 119 for validation).
**Relevance**
This resource is readily useable to train natural language processing tools for predefined clinical and research applications. It can be expanded to extract information on specific drugs, treatment regimens, and adverse effects; and the algorithm and tools can be adapted to most solid tumors for which therapy responses are evaluated by RECIST.

RECIST^4^ is a set of widely accepted rules to define tumor response for patients undergoing antineoplastic therapy. The majority of clinical trials evaluating cancer treatments for objective response in solid tumors use RECIST, which was developed and published in 2000 (version 1.0) and subsequently updated in 2009 (version 1.1), defining four levels of objective tumor response for antineoplastic therapies: complete response (CR), partial response (PR), stable disease (SD), and progressive disease (PD). The RECIST definition of pharmacotherapy response has been widely used for cancer clinical and epidemiologic studies.^5,6^ Despite wide use for clinical trial end points, documentation of formal RECIST evaluation and results are less common in nonclinical trial, real-world settings.^7^ Moreover, manual extraction of RECIST information from EHRs is time consuming; thus, informatics tools that can automatically extract RECIST are highly desirable.

Our ultimate goal is to develop NLP tools that can automatically extract an approximation of cancer treatment and outcome information from clinical notes, because much of such information is embedded in narrative documents in EHRs. Nevertheless, two challenges exist for developing RECIST NLP tools: (1) it is not clear how RECIST information is stored in EHRs and (2) there is a lack of standard annotated corpori for RECIST information extraction. To address these challenges, we conducted this study to identify textual sources for RECIST information in EHRs and to develop a corpus of pharmacotherapy and response entities for development of NLP tools.

Although our current work focuses on predefined treatments as a single group, “pharmacotherapy,” the gold standard data set with built-in training and validation cases established is readily expandable to specific drugs and regimens; moreover, the RECIST tools to be developed on the basis of the data set will be applicable to solid tumors.

## METHODS

### Assembly of a Gold Standard Data Set

The patient base for the data set is an existing, 20-year, lung cancer cohort established in the Mayo Clinic Epidemiology and Genetics of Lung Cancer database.^8-10^

#### Inclusion.

**FIG 1.**
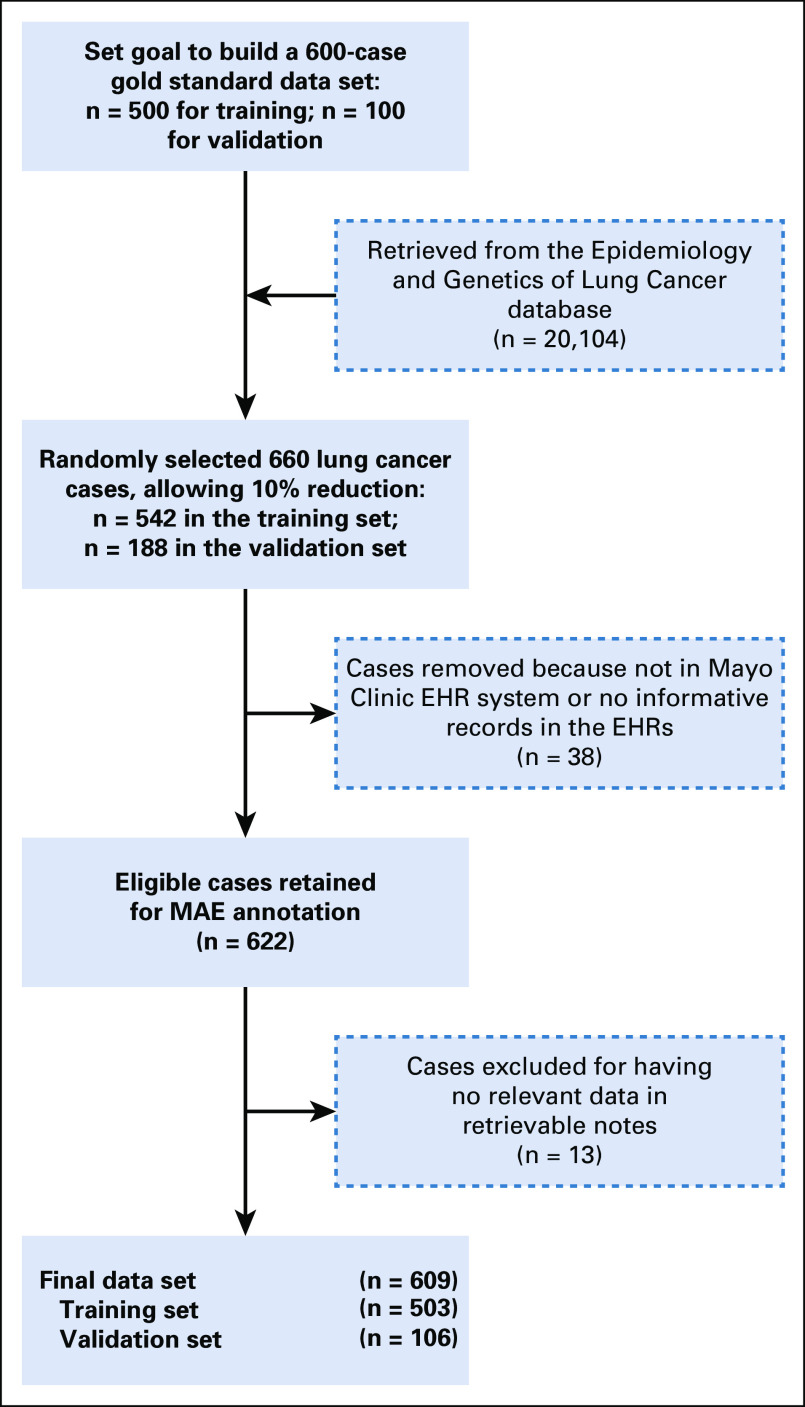
We randomly selected 660 patients, aiming to achieve 600 in the gold standard data set. A final total of 622 patients met the inclusion criteria for the study. The resulting data set comprised 609 cases, 503 for training and 106 for validation purposes. EHR, electronic health record; MAE, Multidocument Annotation Environment.

All patients were aged ≥18 years and were selected from a previously described, large cohort of patients with primary lung cancer.^8-10^ Eligible patients had lung cancer diagnosis confirmed by histopathology or cytology and were treated at Mayo Clinic. Patients were treated with at least one cycle of one or more antineoplastic drugs and had a measured drug response. All patients consented to participate in the study or had given authorization to allow their medical record information reviewed for research. Patients without antineoplastic drug exposure and with incomplete EHRs were not eligible for the current study, as shown in Figure 1. Noted is the time window between 1997 and 2000, when Mayo Clinic transitioned from paper medical records to EHRs, with an anticipated 10% incomplete conversion. We identified all patients with primary lung cancer who received antineoplastic drug therapy at Mayo Clinic with a known response. A random selection of 660 patients from this cohort was assigned to the training set (n = 542) or the validation set (n = 118); our aim was to achieve 600 total cases in the data set. A final total of 622 patients met the inclusion criteria for the study (Fig 1).

### Identifying Sources of RECIST Information

#### Key terms annotation procedure.

The categorization of systemic therapy by intent and possible responses are described in Table 1. For palliative, adjuvant, and induction pharmacotherapy, we evaluated patients for CR, PR, SD, and PD. Although consolidation and maintenance pharmacotherapy deepens responses in some studies, for the purposes of this analysis, we only evaluated for disease stability or progression during this phase of care.

**TABLE 1.**
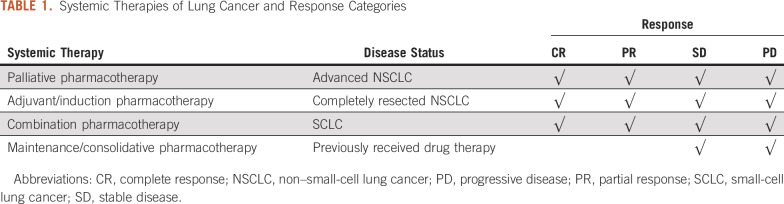
Systemic Therapies of Lung Cancer and Response Categories

#### Note types within Mayo Clinic EHRs.

The Mayo Clinic EHR system contains at least 31 types of clinical documentation, which are described in the Data Supplement. The purpose of this study was to extract response data from unstructured clinical documentation; thus, we abstracted only the clinical documents relevant to this analysis. These are labeled in the Mayo EHR as Subsequent Visit (SV), Consult (CON), Miscellaneous (MIS), and Summary (SUM). There are many other conditions that are described within the EHRs that are common in patients with lung cancer and that may have relevance in the selection of therapy. These conditions were not currently annotated but can be extracted feasibly from the same documents used for the current work focusing on response assessment. We divided the conditions into three categories: comorbid diseases (comorbidity), drug-induced adverse conditions (drug-induced), or cancer-related symptoms not contributing to evaluation of RECIST responses to antineoplastic drugs (eg, symptomatic hypercalcemia).

### Annotating RECIST Entities for Corpus Development

#### Annotation guidelines.

Using an iterative approach, we developed guidelines for annotating RECIST information. Definitions of entities and attributes are described in the following paragraphs.

##### Definition of RECIST response.

Taking as reference the smallest sum of diameters of target lesions, there are four response levels: (1) CR: Disappearance of all target lesions; any pathologic lymph nodes (whether target or nontarget) must have reduction in the short axis to < 10 mm. (2) PR: At least a 30% decrease in the sum of diameters of target lesions, taking as reference the baseline sum diameters. (3) PD: At least a 20% increase in the sum of diameters of target lesions (including baseline sum) and an absolute increase of at least 5 mm. The appearance of one or more new lesions is also considered progression. (4) SD: Neither sufficient shrinkage to qualify for PR nor sufficient increase to qualify for PD. Annotation of RECIST response was based on information found in the clinical notes summarizing the imaging findings, without the raw imaging read by coders themselves.

##### Definition of pharmacotherapy.

**FIG 2.**
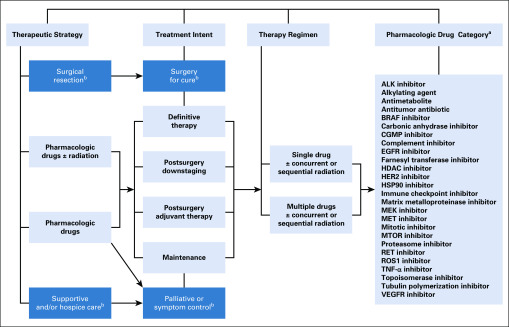
There are four major dimensions of antineoplastic treatment of primary and/or recurrent tumors: therapeutic strategy, treatment intents, therapy regimen, and pharmacologic drug category. (^a^) Alphabetically for coding search convenience. (^b^) Not in the scope of current study.

An overview of lung cancer treatments and detailed pharmacotherapy strategies is provided in the Data Supplement. Our current work focuses on predefined treatments as a single group, pharmacologic drugs (Fig 2), and detailed drug information is provided in the Data Supplement. We documented details of alternative terms for pharmacotherapy in EHRs (ie, all encountered variations for therapy and responses) to account for the four RECIST categories. In addition, we also defined three attributes for CR, PR, PD, and SD:

1. Certainty: evidence of pharmacotherapy and evidence of each level of response. Negated: the problem does not exist in the EHRs. Possible: patient may have a problem, but there is uncertainty expressed in the note. Possible takes precedence over negated, so terms like “probably not” or “unlikely” categorize problems as being possible, just as “probably” and “likely” do. Hypothetical: medical problems the note asserts the patient may develop or discuss with. Positive: the chemotherapy or response was confirmed.
2. Status: the tense in which treatment or response occurs: historical or present
3. Exclusion: Key terms used outside of the study scope. Exclude the responses that are not for lung cancer or lung site or pharmacotherapy.

#### Annotation procedure.

##### Deidentification.

We used an in-house pipeline to detect protected health information (PHI) and manual annotation of PHI in parallel by two independent teams; subsequently, the PHI information identified through the pipeline and the manual way were combined and removed.

##### Annotation tool.

Multidocument Annotation Environment (MAE), an annotation tool for natural language text annotation, allows users to define their own annotation tasks flexibly, annotate partial words, use nonconsuming tags, and easily create links between extent entities. It outputs annotations in stand-off XML. Although it does not enforce strict rules for annotation schemas, it is easy to set up and start running.^11^ Although MAE does not represent a new frontier in annotation software, its ease of use, portability, and clean visualization make it useful and efficient for annotation projects.

##### Training and annotation procedures.

Three coders were trained; coders 1 and 2 performed the entire annotation and coder 3 was an adjudicator when disagreement occurred between coders 1 and 2 during a five-step training process using five note sets: set 1: 10 notes (notes 1-10), learning together with a trained coder; set 2: 15 notes (notes 11-25), learning independently, dissecting, and checking; set 3: 11 notes (notes 26-36), coding independently, debating, and converging; set 4: 17 notes (notes 37-53), coding independently, comparing, and refining; and set 5: 28 notes (notes 54-71), coding independently and reaching consensus. Detailed training results are provided in the Data Supplement.

##### Annotated informative clinical notes.

For most cases, several selected clinical notes contained duplicated information and stated the same period of pharmacotherapy or a measured drug response, largely were not directly relevant to antineoplastic therapy, and instead, related to patients’ comorbid conditions and treatment adverse effects or toxicities, as listed in the Data Supplement. To concentrate informative data during annotation, we searched, reviewed, and selected at least one clinical note for each case covering all key terms.

## RESULTS

We randomly selected 622 eligible patients with lung cancer in our database and extracted 55,120 total medical notes (n = 72 types) in our institutional EHR system. After applying the key words “complete response,” “partial response,” “stable,” and “progressive disease,” we reduced clinical notes to 37,406 in 31 types (Data Supplement). Then, using four note types (SV, CON, MIS, SUM) within 12 months after diagnosis further reduced the number of clinical notes to 5,005—9% of the original total (n = 5,005 of 55,120). Most cases (90%) had more than five notes before annotation (Data Supplement). Manual annotation using MAE narrowed 1% of cases to more than four notes; the resulting 736 notes included all key terms and covered 97.9% of all cases (n = 609 of 622); the remaining notes had repeated or irrelevant information to the current work. Detailed annotations of 1.3% of the total clinical notes (n = 736 of 55,120) provided essential data for 97.9% of all eligible cases. More specifically, we found 13 cases (n = 11 in the training set and n = 2 in the validation set) of 622 cases had no informative key terms in the retrieved clinical notes (Data Supplement); specifically, there were five cases without response, four cases without drug information, and four cases with neither response nor drug information. Various RECIST categories were applicable depending on specific therapeutic strategies (Table 1). For example, when patients received maintenance or consolidative pharmacotherapy, their RECIST responses would be SD or PD.

The resulting data set of 609 cases (n = 503 for training and n = 106 for validation purposes) and the two subgroups can be flexibly reassigned. The 736 fully annotated, deidentified clinical notes formed a data corpus with pharmacotherapies and the four response levels (CR, PR, SD, and PD). A basic description of demographic and clinical information of the data set cases is provided in Table 2. Of the total 609 patients, the mean age at the time of diagnosis was 64.3 years (range, 31.7-89.3 years); median follow-up time is 18.1 months (range, 1.2-242.4 months); non–small-cell lung cancer was the most frequent lung cancer subtype; and there is no significant difference in each variable between the two cohorts.

**TABLE 2.**
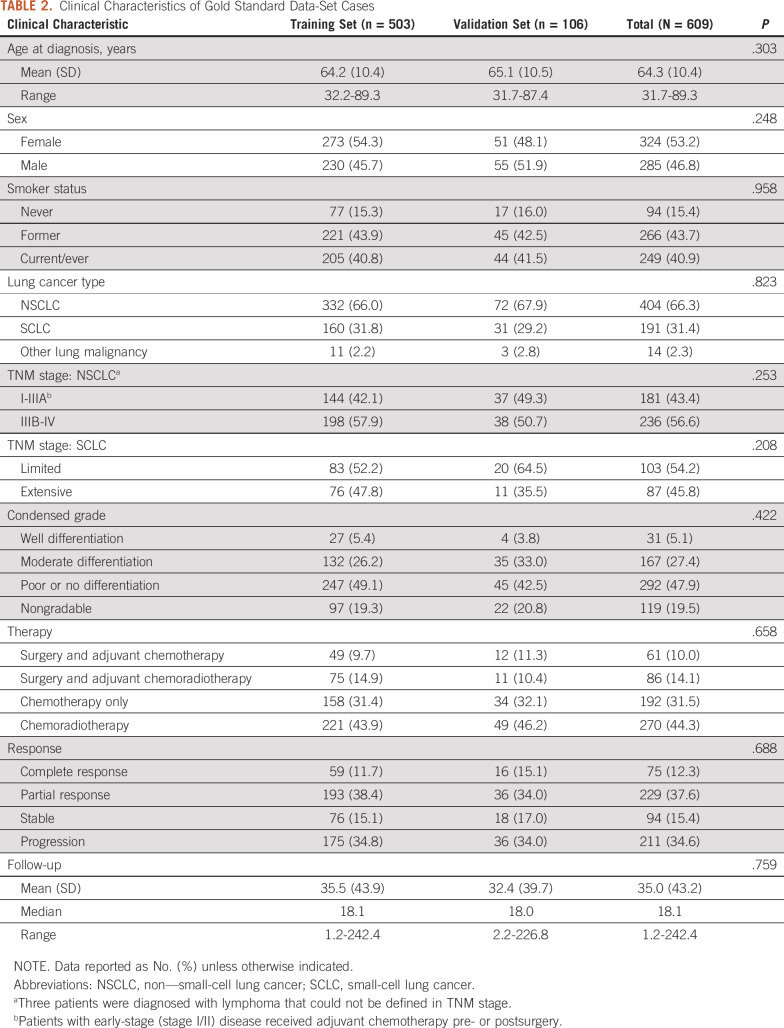
Clinical Characteristics of Gold Standard Data-Set Cases

Detailed therapy regimens and specific drug combinations are available in the data set, as seen in Table 3, which illustrates the initial treatment drugs and combinations of the data set. Nearly 80% of the patients received doublet therapies, mainly with a platinum compound and another drug. Such information for subsequent treatment is also available (data not shown).

**TABLE 3.**
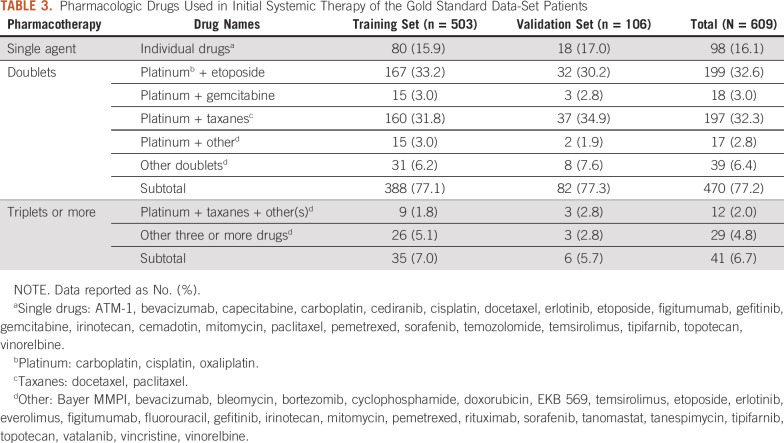
Pharmacologic Drugs Used in Initial Systemic Therapy of the Gold Standard Data-Set Patients

Working guidelines for searching and annotating key terms and attributes are provided in Table 4, which documented encountered alternative terms for “pharmacotherapy” in EHRs, which varied from “therapy” in the context of anticancer drug names, chemotherapy, specific drug names (eg, “etoposide and cisplatin,” “paclitaxel,” “tarceva,” “gemcitabine”). Variations in four attributes are also documented. Data Supplement Table 7 summarizes Other conditions recorded in the clinical notes of the data set cases as summarized in the Data Supplement; some are under more than one category because they could result from different causes.

**TABLE 4.**
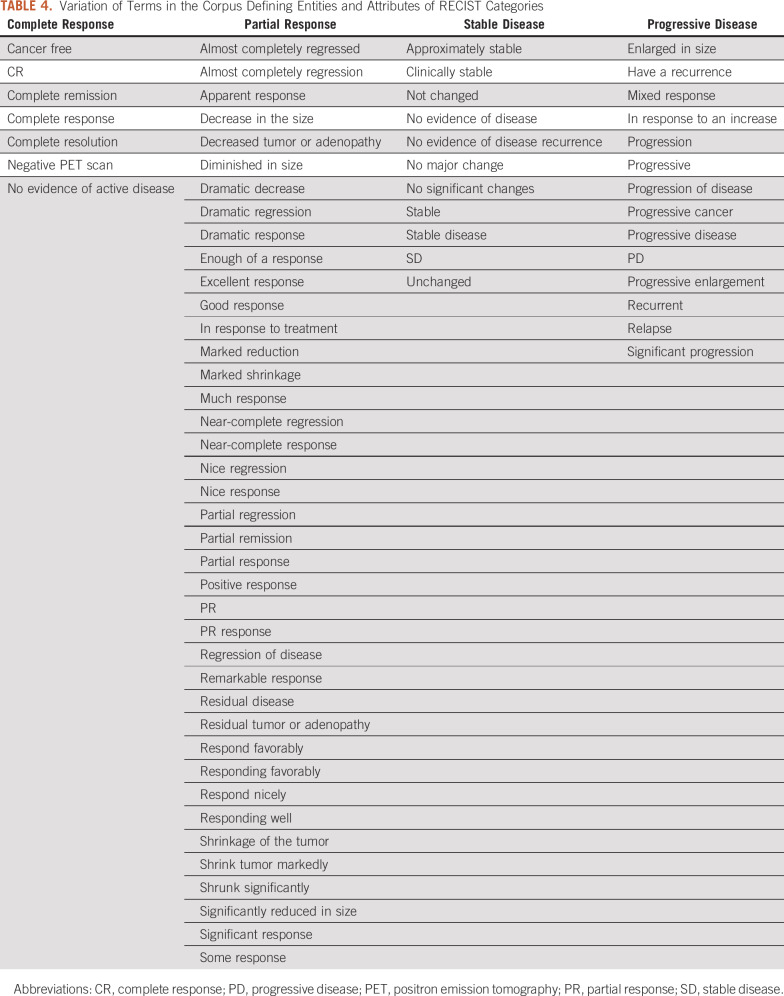
Variation of Terms in the Corpus Defining Entities and Attributes of RECIST Categories

The fully annotated data set will be released upon request to academic and noncommercial users. Although great efforts have been made to fully deidentify the data set, appropriate data use agreements will still be required.

## DISCUSSION

We have created a gold standard data set of 609 cases, with built-in training and validation sets, to effectively capture all relevant information contributing to response levels as defined by RECIST. We demonstrated how to effectively reduce the seemingly insurmountable numbers of clinical notes: that is, 55,120 medical notes in 72 types, recorded in Mayo Clinic’s EHRs, reduced to 736 that covered 609 patients—specifically, annotations of 1.3% of total clinical notes provided essential information for 97.9% of all eligible cases. Our objective was to support an expedited process of extracting antineoplastic therapy response information in a real-world setting of patients diagnosed with solid tumors, exemplified through lung cancer, with a specific goal to develop an NLP-supported RECIST tool for extracting and synthesizing antineoplastic therapy responses from EHRs in accelerating research into antineoplastic therapeutic response. Tools built from this resource could accelerate standardized evaluation of patient outcomes in a wide scope and is applicable to different drug groups and specific regimens of virtually all solid tumors when using RECIST.

Notably, there were several limitations in this study. One is that we only extracted the responses and all drug regimens combined. However, there were several confounding variables between the response and pharmacotherapy. For example, the relevance of response to multiple lines of chemotherapy, the various drugs, treatment of comorbidity and other conditions, surgical radicality, and adjuvant therapy, which caused complexity in the comparison. In our current study, we only selected the pharmacotherapy and response level as defined by RECIST. Because of heterogeneity of treatments and responses (eg, CR is unusual in metastatic non–small-cell lung cancer), the corpus has class imbalances that may limit generalizability to all clinical scenarios.

Another limitation is that we emphasized antineoplastic drugs, not including other treatment modalities. In real-world practice, there are diverse treatments for lung cancer, including surgery, drug therapy, radiation therapy, and palliative care. RECIST may be applicable to radiation therapy and palliative care interventions.

In terms of future directions and ongoing efforts, we are developing an NLP tool to accurately extract RECIST-based treatment responses in patients with cancer. Despite the widespread adoption of EHRs and multiple efforts to rapidly retrieve useful information to improve patient care, researchers remain challenged by the heterogeneity of EHRs data. Much of the information required to conduct precision medicine, which encompasses the individualized capture of responses to treatment, is contained within unstructured, written texts and clinical notes. In its current state, this information is not computable; hence, NLP offers an exciting opportunity to unlock these data. Menasalvas Ruiz et al^12^ reported in 2018 on a first integration of an NLP framework for the analysis of clinical records of patients with lung cancer, making use of a telephone assistance service of a major Spanish hospital. The study specifically showed how relevant data (ie, patient demographics and medical comorbidities) can be extracted, and how these data can be used to conduct relevant analyses. Their study demonstrates that integration of unstructured EHR text within a data analysis framework is technically feasible and worthy of additional study. However, to our knowledge, there is no NLP system capable of extracting antineoplastic treatment response from EHRs. In fact, large commercial entities such as Flatiron Health (New York, NY) have specifically identified this as a highly challenging area.^7,13,14^ The aim of our NLP tool is to quickly and easily enable response extraction for lung cancer pharmacotherapy in oncology and for additional medical questions, which can expand to specific antineoplastic drugs and extend to all solid tumors.

Our gold standard data set contains comprehensive information on each individual record that can be used for additional purposes beyond determining RECIST-defined responses. For instance, our framework can extract each line of treatment, patient comorbidity, toxicity/adverse event, or relapse/progression that occurs, as proposed in the following paragraphs.

Some treatment attributes were not retrieved due to responses being described imprecisely in clinical documentation. A goal is to link treatment responses to specific therapy regimens.

As depicted in Figure 1, treatment options for lung cancer include surgery, radiation therapy, chemotherapy, immunotherapy, and targeted therapy. Therapeutic-modality recommendations depend on several factors, including the type and stage of cancer. In this study, we only focused on pharmacotherapy and analyzed drug exposures regardless of stage. In future research, we will categorize drug exposures on the basis of treatment intent.

Treatment toxicities can vary significantly depending on the class of pharmacotherapy received. Distinguishing the toxicities associated with different classes of therapies on the basis of EHR data will be a challenging but crucial focus of future work.

In our current study, we only focused on evaluation of target lesions in the thorax. RECIST criteria can also be applied in evaluation of nontarget lesions in metastatic sites as well as pathologic lymph nodes.

Patients with lung cancer often have multiple comorbidities. The overall impact of preexisting conditions on lung cancer outcomes is not known and can be difficult to determine. Others have shown comorbidities predict overall survival independently in response to antineoplastic therapy, although the effects are often modest.^15,16^ Extraction of comorbidity data captured within the EHRs and correlation of these data with long-term outcomes are a compelling focus of future work.
